# Starving the enemy: how plant and microbe compete for sugar on the border

**DOI:** 10.3389/fpls.2023.1230254

**Published:** 2023-08-02

**Authors:** Jingsheng Chen, Miao Sun, Guosheng Xiao, Rujie Shi, Chanjuan Zhao, Qianqian Zhang, Shuo Yang, Yuanhu Xuan

**Affiliations:** ^1^ College of Biology and Food Engineering, Chongqing Three Gorges University, Wanzhou, China; ^2^ Chongqing Three Gorges Vocational College, Wanzhou, China; ^3^ College of Plant Protection, Shenyang Agricultural University, Shenyang, China

**Keywords:** host-pathogen interaction, microbe, sugar transporters, sugar competition, plant immunity

## Abstract

As the primary energy source for a plant host and microbe to sustain life, sugar is generally exported by Sugars Will Eventually be Exported Transporters (SWEETs) to the host extracellular spaces or the apoplast. There, the host and microbes compete for hexose, sucrose, and other important nutrients. The host and microbial monosaccharide transporters (MSTs) and sucrose transporters (SUTs) play a key role in the “evolutionary arms race”. The result of this competition hinges on the proportion of sugar distribution between the host and microbes. In some plants (such as *Arabidopsis*, corn, and rice) and their interacting pathogens, the key transporters responsible for sugar competition have been identified. However, the regulatory mechanisms of sugar transporters, especially in the microbes require further investigation. Here, the key transporters that are responsible for the sugar competition in the host and pathogen have been identified and the regulatory mechanisms of the sugar transport have been briefly analyzed. These data are of great significance to the increase of the sugar distribution in plants for improvement in the yield.

## Introduction

The contradiction between the growing population’s demand for food and the safety of food production is becoming increasingly severe. Furthermore, plant diseases continue to seriously threaten the safety of food production worldwide (Strange and Scott, 2009). The global yield loss due to plant diseases and insect pests in different crops including potato, soybean, wheat, maize, and rice can reach 17.2%-30.3% annually (Ristaino et al., 2021). To facilitate the reduction of food loss caused by plant diseases and maintain food security, future studies should focus on identifying the QTLs of partial and persistent resistance of plants and the relatively conservative pathogenic factors, including pathogen-associated molecular patterns (PAMPs) and effectors, in the pathogenic bacteria from the level of host-pathogen interaction, the disease-resistant gene as one of the effective tools of disease management will be exchanged and managed in a wide range (Boyd et al., 2013; Garrett et al., 2017). Therefore, to identify effective and stable resistance genes, the key factors involved in the competition between pathogens and hosts are of significant interest.

## Photosynthetic sugar is the key nutrient for plants and microbes

The primary competitive nutrients between plants and pathogens, such as sugars, may become significant factors in disease control. Sugar, as the main source of energy, is vital for plants and pathogens and involved in regulating different physiological functions and metabolic pathways, including growth regulation, signal transduction, structural component synthesis (i.e., carbon skeleton), and osmotic pressure (Rolland et al., 2006; Saddhe et al., 2021). Although fatty acids, as organic carbon sources for fungi, play a significant role in fungal symbiosis, sugars also function as precursors or nutrients in both plants and pathogens(Luginbuehl et al., 2017; Bouwmeester, 2021). As a producer, plants synthesize carbohydrates that are transported from ‘source’ to ‘sink’ by sugar transporters in the form of sucrose *via* photosynthesis and assimilated in the mesophyll (Julius et al., 2017). During the invasion process, pathogenic bacteria must secure sugar from the plant host to supply their own growth. To capture more sugar, the pathogen must force the host plant to transport the sugar (e.g., glucose, fructose, or sucrose) from within the cell to the apoplast, a frontline where the host and the pathogen can interact with each other. This process requires the participation of the plasma membrane-localized sugar transporters (Julius et al., 2017) (Naseem et al., 2017).

## The host sugar transporter is hijacked by the microbes to export sugar from the cell

**Figure 1:**
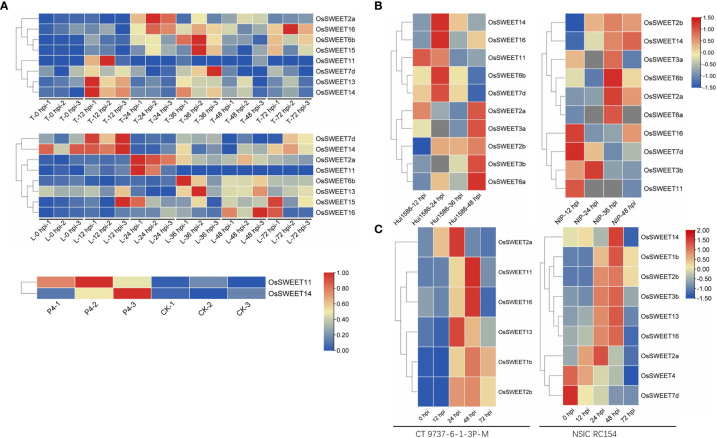
Heat map of the expression pattern of the differentially expressed *SWEET* genes in the rice transcriptome induced by four pathogens. **(A)** Gene expression at 0, 12, 24, 48, and 72 h post-inoculation with *R. solani* AG1-IA in the resistant Teqing (T) or susceptible Lemont (L) rice lines and the FPKM at 9 days post-inoculation with *Ustilaginoidea virens* P4 strain in susceptible Pujiang6 rice variety. Every three columns marketed by 1, 2, or 3 correspond to a time or a treatment result (Fan et al., 2020; Wang et al., 2021). **(B)** log2 fold gene expression at 12, 24, 36, and 48 h post-inoculation with *Magnaporthe oryzae* Guy11 strain in the resistant Hui1586 or susceptible Nipponbare (NIP) rice lines (Yang et al., 2021). **(C)** Gene expression at 0, 12, 24, 48, and 72 h post-inoculation with *Xanthomonas oryzae* pv. oryzae P3 and P6 strains in resistant NSIC RC154 or susceptible CT 9737-6-1-3P-M lines (Shu et al., 2021).

**Figure 2:**
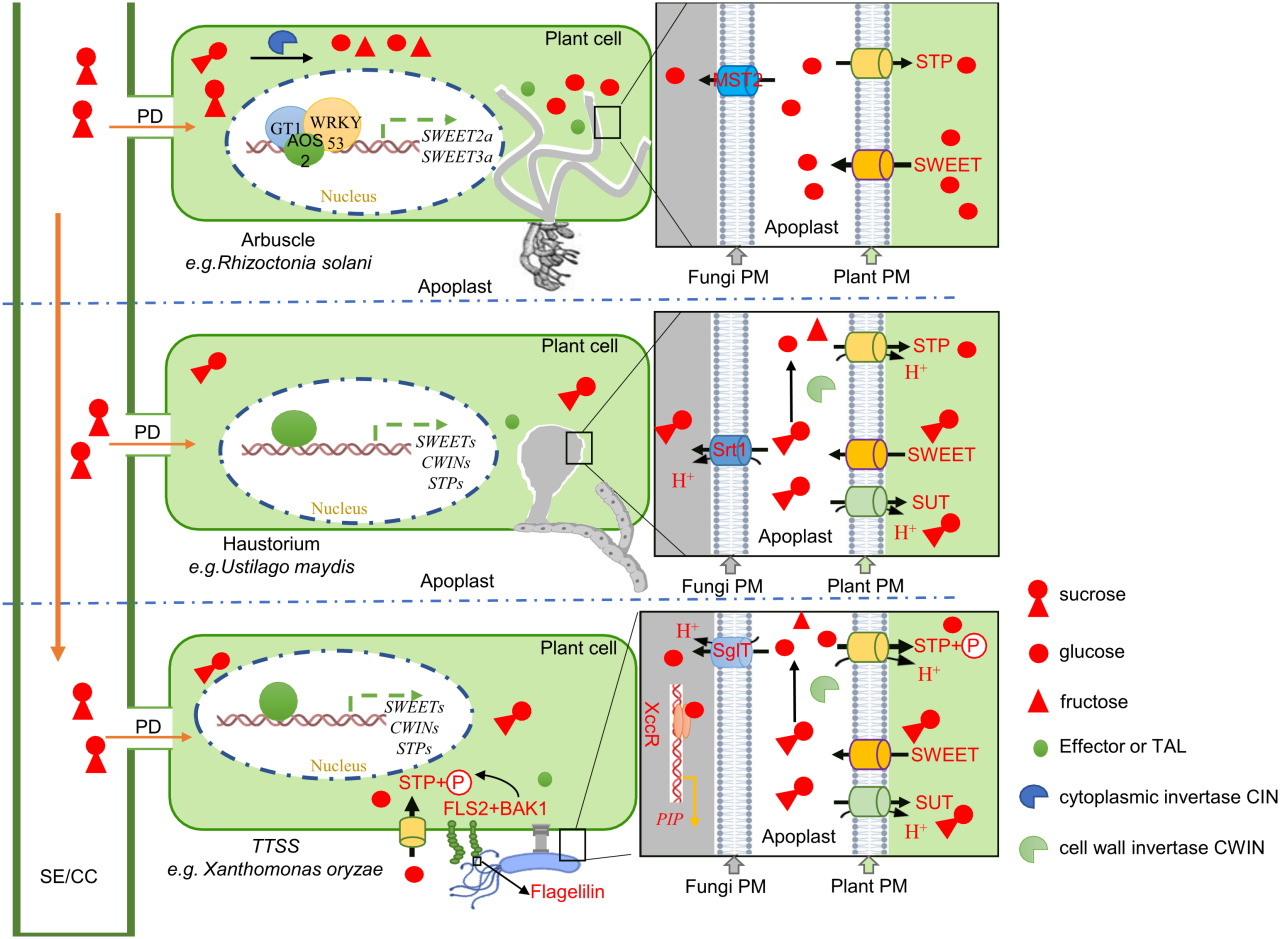
An illustration demonstrating the putative mechanism of sugar transporter regulated by a signal or regulator molecule from different microbes during host-pathogen interaction. To meet the demand for sugar, microbes directly or coupled with transcription factors (TF) such as WRKY53 or GT1, activate the *SWEET* genes in the plant that mediate glucose or sucrose efflux *via* effector protein (AOS2) or transcription activator-like (TAL) (e.g., PthXO1, TALC, and pthAw) effector secretion into the host (Yang et al., 2006; Antony et al., 2010; Liu et al., 2011; Hu et al., 2014; Yang et al., 2022). However, CWIN in plants is also activated to hydrolyze the sucrose in the apoplast increasing the content of extracellular hexose and causing the host and pathogen to compete for hexose *via* hexose transporter (STP/H^+^ in plants and MST/H^+^ or SglT/H^+^ in microbes) (Voegele et al., 2001; Helber et al., 2011; Proels and Hückelhoven, 2014; Zhang et al., 2023). Following microbial absorption, hexose (especially glucose) is perceived by the LuxR receptor as a signal molecule, leading to the activation of an important pathogenic protein (PIP) (Zhang et al., 2023). Similarly, glucose also mediates the activation of the host immune response when sugar leakage occurs in the host-microbe interaction interface (Schuler et al., 2015). In addition, the host FLS2 responds to bacterial flagellin and interacts with BAK1 to phosphorylate STP to accelerate hexose absorption by the host(Yamada et al., 2016). This restriction is relieved by the nutrition strategy of microbes absorbed by sucrose. Pathogenic bacteria have a greater advantage in competition with low-affinity sucrose transporters (SUTs) in plants *via* high-affinity sucrose transporters (SRT) (Wahl et al., 2010).

Sugars Will Eventually be Exported Transporters (SWEETs) are one of the three known sugar transporter families, including monosaccharide transporters (MSTs) and sucrose transporters (SUTs)(Misra et al., 2019). They are involved in the intracellular and extracellular transport of sugar by pH-independent uniporters and undertake the central role in phloem loading, nectar secretion, seed filling, pollen nutrition, and plant senescence (Chen et al., 2012; Eom et al., 2015). The SWEET protein is composed of α-helix transmembrane domains (TMs) and functions as a sugar efflux carrier by forming oligomers (Xuan et al., 2013). In addition, the SWEET proteins play an important regulatory role in the plant-pathogen interaction (Breia et al., 2021). Within the pathosystem, *SWEETs* function as susceptible (S) genes and are induced during pathogen infection, resulting in increased pathogen virulence (Gupta, 2020; Mondal et al., 2021). The transcriptome data of different rice lines (resistant Teqing (T) and susceptible Lemont (L) were analyzed. The results demonstrated that eight *SWEET* genes (*OsSWEET2a, 6b, 7d, 11, 13, 14, 15*, and *16*) were up-regulated following *Rhizoctonia solani* AG1-IA strain inoculation (Wang et al., 2021) (**Figure 1**); two *SWEET*s (*OsSWEET11* and *14*), of the susceptible Pujiang6 rice line were up-regulated (3.4 and 5.8-fold, respectively) after nine days following the inoculation with *Ustilaginoidea virens* P4 strain (Fan et al., 2020) (**Figure 1A**). Ten *SWEET* genes (O*sSWEET2a, 2b, 3a, 3b, 6a, 6b, 7d, 11, 14*, and *16*) of the resistant Hui1586 and susceptible Nipponbare (NIP) lines responded to *Magnaporthe oryzae* Guy11 strain induction (Yang et al., 2021) (**Figure 1B**). In the susceptible CT 9737-6-1-3P-M rice line, six *SWEET* genes (*OsSWEET1b, 2a, 2b, 11, 13*, and *16*) were up-regulated by *Xanthomonas oryzae* pv. *oryzae* P3 and P6 strains following inoculation. However, in the resistant NSIC RC154 line, seven *SWEET* genes (*OsSWEET1b, 2a, 2b, 3b, 13, 14*, and *16*) were up-regulated following inoculation while two were not (*OsSWEET4* and *7d*) (Shu et al., 2021)(**Figure 1C**). Transcriptomic analysis, as well as the analysis of gene expression and sugar contents, of the inoculated samples has shown that *SWEET*s are up-regulated in response to competition with invading pathogens for sugar. Twenty-one SWEETs have been identified in rice, among which Xa13/OsSWEET11 (targeted by PthXO1) and Os11N3/OsSWEET14 (targeted by TALC) are induced by various transcription activator-like (TAL) effectors secreted *via* type III secretion system of *Xoo*, which cause bacterial leaf blight. Xa25/OsSWEET13 has been shown to be induced by *Xoo* PXO339 strain as a susceptible allele (Yang et al., 2006; Antony et al., 2010; Liu et al., 2011). SWEET-mediated susceptibility dependence on TAL effector also occurs in other plants, where *GhSWEET10* in cotton is specifically activated by an Avrb6-mediated effector (Cox et al., 2017) and *CsSWEET1* in citrus is activated by effector pthAw or pthA4 from the *X. citri* strain (Hu et al., 2014). Streubel et al. identified that OsSWEET12 and 15 are induced by artificial TALs to promote the distribution of sugar flow (primarily sucrose) in pathogens (Streubel et al., 2013). However, SWEET-targeting has been reported in other bacteria and fungi. OsDOF11 (DNA BINDING WITH ONE FINGER 11) regulates the expression of *OsSWEET11*, *OsSWEET14*, and *OsSUT1* by directly binding to the gene promoter. Furthermore, the overexpression of DOF activates *SWEET14* gene expression, therefore improving rice resistance to sheath blight caused by the invasion of *R. solani* AG1-IA (Wu et al., 2018; Kim et al., 2020). Moreover, Gao et al. determined that *R. solani* promotes sugar efflux and increases pathogenicity by activating *SWEET11* (Gao et al., 2018). Recent research has demonstrated that the infection by the rice blast fungus may activate the BR signal *via* WRKY53 and induce *SWEET2a* expression *via* negative regulation of rice ShB resistance (Gao et al., 2021). Further research identified that the effector AOS2 secreted by *R. solani* interacts with WRKY53 and GT1 to form a transcription factor complex, activating the *SWEET* genes (including *SWEET2a* and *3a*) and increasing the virulence of fungi through nutritional competition (Yang et al., 2022). During the symbiosis of *Medicago truncatula* and arbuscular mycorrhizal (AM) fungi, MtSWEET1b is also induced to promote a glucose efflux carrier (An et al., 2019). Phloem loading is a central link to the long-distance transport of sugars in plants, where SWEET-mediated sugar efflux carriers transport sugars from phloem parenchyma cells to the apoplast. Then, the sugars are transferred into sieve tubes and companion cells *via* SUC, a sucrose carrier (Ruan, 2014). Plant *SWEET* genes are systematically divided into four subclasses. Clades I, II, and IV primarily contain SWEET genes that are monosaccharide efflux transporters that transport glucose, galactose, and/or fructose. However, Clade III members preferentially transport sucrose (Chandran, 2015). The discovery that pathogens with different types and sugar requirements alter the expression of plant SWEET transporters demonstrates that pathogens use a common strategy to extract sugar from the host (Schüßler et al., 2006; Wahl et al., 2010). The strategies used by pathogens to target plant SWEETs differ according to their sugar preferences, and multiple SWEETs in different clades of the SWEET family have been found to be targeted. Therefore, SWEET genes play a primary role in sugar efflux and have a positive effect on microbial pathogenesis (**Figure 2**).

## Host immunity and sugar recovery are activated following response to sugar leakage

With the pathogen hijacking SWEET in the host to expel sugar to the apoplast for its growth, the plant also made a counterattack. In plants and pathogenic bacteria, invertases (INV) are divided into the following categories according to subcellular localization and cleavage of sucrose into fructose and glucose: cell wall invertase (CWIN), cytoplasmic invertase (CIN), and vacuolar invertase (VIN). CWIN plays a key role in the production of crops, particularly in the development of seeds and fruits (Kocal et al., 2008; Ruan, 2014). *GIF1* (*GRAIN INCOMPLETE FILLING 1*) encoding a CWIN is a putative domestication gene, which is required for carbon distribution at the early stage of grain filling in rice (Wang et al., 2008). Furthermore, the activity and expression of CWIN are induced during a pathogen invasion in a plant. Therefore, CWIN is key invertase regulating sugar, specifically sucrose metabolism during plant defense response and is responsible for the hydrolysis of sucrose into fructose and glucose in the apoplast. These hexoses are further recovered by sugar transport proteins in the host and function as signal molecules in the sugar signal pathway (Proels and Hückelhoven, 2014; Liao et al., 2020). Following the infection with *Xanthomonas campestris* pv. *vesicatoria* in tomato, the increased CWIN activity induced by pathogenic bacteria causes the increase of hexose formation, which may further trigger increased negative regulation of photosynthesis and photosynthetic gene expression and positive regulation of sugar-induced pathogenesis-related genes (Kocal et al., 2008). Reduced photosynthesis and improved basic defense response levels are accompanied by reduced sugar outflow and high resistance of the host.

To respond to the microbe-induced sugar leakage, plants can also “starve the pathogens to death” by retrieving sugars (mainly sucrose and glucose) from apoplasts *via* sugar transporters. In plants, along with *SWEET* genes, monosaccharide transporters (MST) (generally including the sugar transporters protein (STP)) also play an important role in sugar transport, specifically in the process of phloem loading and unloading, pathogen defense, and yield formation (Doidy et al., 2012; Li et al., 2018; Zhang et al., 2019) The MST family is subdivided according to sequence differences and substrate specificities into the STP, PLT (polyol/monosaccharide transporter), VGT (vacuolar glucose transporter), INT (inositol transporter), TMT (tonoplast monosaccharide transporter), pGlcT (plastidic glucose translocator), and ERD (early response to dehydration 6-like) subgroups (Büttner, 2007; Deng et al., 2019).

**Figure 3:**
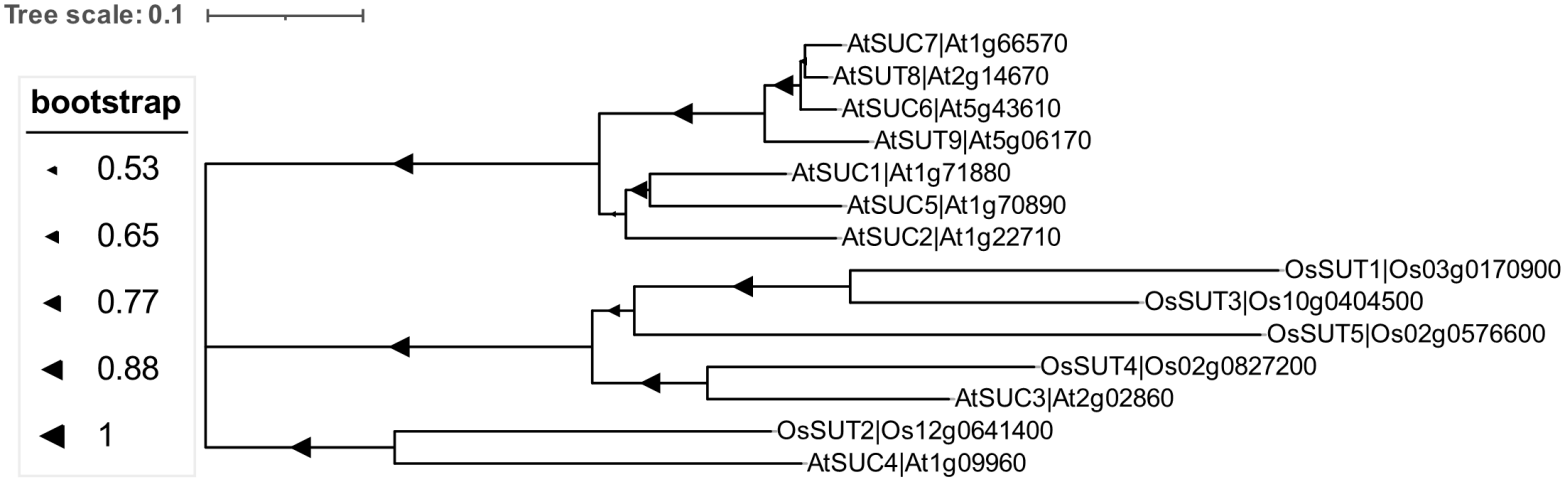
Phylogenetic tree of the sucrose transporters (SUTs) in monocotyledonous plants (*Oryza sativa*) and dicotyledonous plants (*Arabidopsis thaliana*). Multiple sequence alignment analysis of sucrose transporters was conducted by Cluster W (version 2) and the phylogenetic evolution tree was constructed using the Neighbor-Joining (NJ) method using MEGA7.0 software. The phylogenetic analysis was performed using 1,000 bootstrap reiterations with the Jones-Taylor-Thornton (JTT) model.

SUT is a sucrose-H^+^ symporter and is characterized into five groups: SUT1 to SUT5. SUT1 is present in a specific branch of dicotyledonous plants and members of SUT2 and SUT4 branches are found in both monocotyledonous and dicotyledonous plants (**Figure 3**). SUT is expressed in the source leaves and sink cells, and participates in the process of phloem loading and unloading, and responds to dehydration, photosynthesis, circadian rhythm, and the development of nodules (Misra et al., 2019). In the phloem loading process, sucrose is discharged into the apoplast *via* the SWEET family sugar transporters in the sink cells and then absorbed or loaded into the phloem by SUT or SUC sugar transporters. Sucrose transported *via* the companion cells-sieve elements (CC-SE) complexes will eventually be exported or unloaded into the phloem apoplasmically by SWEETs and then absorbed into the sink cells by the action of sucrose/H^+^ cotransporter. Alternatively, this sucrose can be hydrolyzed by the CWIN to produce hexose and then enter the surrounding source cells *via* the hexose transport protein (Braun, 2022). OsSUT1 plays a role in seed filling, germination, and early seedling growth, but is also responsible for sucrose recovery from plastids along the transport path during phloem loading in rice (Scofield et al., 2007). TaSUT1 also functions to recover leaked sucrose from phloem apoplast (Aoki et al., 2014). The source-sink distribution of sucrose plays a key role in regulating plant yield development (Chen and Ham, 2022). Therefore, SWEET-SUT regulates the long-distance transportation of sugars to regulate yield rather than immunity.

For monosaccharide-dependent pathogenic bacteria, plants can recover hexose from the extracellular spaces *via* highly efficient and plasma membrane-localized monosaccharide transport proteins to inhibit the loss of sugar. Similar to SUT, as a member of the major facilitator superfamily, MSTs contain 12 transmembrane domains linked by hydrophilic loops and have the ability of H^+^-sugar cotransporters. STP, as a member of the monosaccharide transporters family, absorbs broad-spectrum monosaccharide substrates with high affinity and retrieves hexose from the plant extracellular spaces, especially when challenged by microbe (Büttner, 2010; Deng et al., 2019; Sosso et al., 2019). In *Arabidopsis*, the widespread biotrophic pathogen *Erysiphe cichoracerum* significantly induces *AtSTP4* gene expression following inoculation. The pathogenic fungus *Botrytis cinerea* causing grey mold disease induces *AtSTP13* gene expression to enhance the level of glucose absorption of plants and improves plant resistance to *Botrytis cinerea* (Fotopoulos et al., 2003; Lemonnier et al., 2014). Furthermore, *AtSTP13* is phosphorylation-dependent and functions as a positive regulator of disease resistance in *Arabidopsis*. In the presence of pathogenic bacteria, flagellin sensitive 2 (FLS2), the pattern recognition receptors (PRRs) expressed on the cell surface, and BRASSINOSTEROID INSENSITIVE 1–associated receptor kinase1 (BAK1) interacts with STP13 by phosphorylating it at the threonine 485 site, resulting in improved hexose absorption capacity by the plant. The pathogenicity of bacteria is therefore weakened by limiting the nutrient absorption from the host (Yamada et al., 2016). Therefore, SUT and STP located on the plant plasma membrane absorb sucrose into the sink tissue and retrieve monosaccharides from the apoplast to reduce sugar loss (**Figure 2**). These sugar transporter functions are instrumental to the development of plant yield and the improvement of resistance.

## Microbes scramble for sugar to survive at the conflict border

The ability to utilize sugars in plant tissues promotes the intensity of the synthesis of photosynthesis products to a certain extent (Braun et al., 2014). Following the invasion of a pathogen, the increased sugar utilization in plant tissues also causes increased sugar flow to the pathogen, resulting in the loss of yield (**Figure 2**) (Pommerrenig et al., 2020). The glomeromycotan fungus (*Geosiphon pyriformis*) monosaccharide transporter GpMST1 is a proton cotransporter and contains a very low GC content and has the highest affinity for monosaccharides, including glucose, mannose, galactose, and fructose (Schüßler et al., 2006). Due to the hydrolysis of sucrose by plant CWIN at the host-microbe interaction interface numerous glucose and fructose molecules are present in the apoplast. In order to avoid the immune defense reaction caused by the activation of plant CWIN, the microbes also encode their own CWIN (Doidy et al., 2012). The invertase UfINV1 of *Uromyces fabae* rust fungus has been identified and has been found to disturb sugar partitioning during host-pathogen interaction (Voegele et al., 2007). Pathogens can directly absorb these hexoses to maintain growth and toxicity. In *B. cinerea*, a gray mold pathogen, a high-affinity proton symporter FRT1 transports fructose specifically and plays a key role in the process of vegetative growth and pathogenesis (Doehlemann et al., 2005). Another high-affinity monosaccharide transporter MST2 expressed on symbiotic hyphae of *Rhizophagus irregularis* mediates sugar absorption from the interface of *Glomus*-*Medicago truncatula* interaction (Helber et al., 2011). *U. fabae* obtains sugar from *Vicia faba via* haustoria, where concentrated gene expression encoding hexose transport proteins, including HXT1, specifically combines the substrate D-glucose and D-fructose (Voegele et al., 2001). HXT1 has a high substrate affinity for glucose, fructose, and mannose. The hexose absorption capacity and toxicity of the microbe are therefore significantly weakened with an HXT1 mutation. The heterologous expression of CgHXT3 and AtSTP1, which have high-affinity hexose transporter comparable to HXT1, fail to repair the pathogenicity defect, suggesting that HXT has dual functions of a hexose receptor and transporter during the plant invasion process (Schuler et al., 2015). In line with this, pathogen invasion as an exogenous stress activates CWIN (AtCWINV1) and SWEET (AtSWEET2 and 15) genes in plants, resulting in glucose accumulation in the infected plastids. The glucose absorbed by the sugar transporter SglT of *Xanthomonas campestris* pv. *campestris* (*Xcc*) combines with the orphan governor XccR, the homologous protein of cognate receptors LuxR, to activate the proline aminopeptidase-encoded gene *PIP* and enhance virulence (Zhang et al., 2023). Directly-absorbed and abundant hexose, represented by glucose, is the necessary nutrient and signal molecule for the pathogen. Hexose-dependent nutrition strategy is limited by what plants have evolved as a defense mechanism to reprogram immune-related genes in response to the changes in glucose content at the infection sites (Ehness et al., 1997).

However, the strategy of reducing energy to limit microbial virulence is overcome by pathogens with sucrose as the primary carbon source (Talbot, 2010). Sucrose, as the major product of photosynthesis and the main form of long-distance transport, is very abundant in plants. This kind of pathogen avoids utilizing the invertase to cleave sucrose to hexose in the extracellular spaces and avoids the recognition and activation of plant defense reactions, including active oxygen burst and cell death. The sucrose transporter (Srt1) is localized on the plasma membrane of *Ustilago maydis*, which causes corn smut. Srt1 has extremely high substrate affinity and specificity for sucrose, outperforming plant sucrose transporters (such as SUT) with low sucrose affinity and energy dependence to absorb sucrose from the apoplast of the host directly and avoids the plant immune response triggered by monosaccharide leakage (Talbot, 2010; Wahl et al., 2010). This strategy of pathogenic bacteria increases the sugars in the apoplast flow in the form of sucrose to support its own virulence and growth requirements.

## Conclusions and future prospects

**Figure 4:**
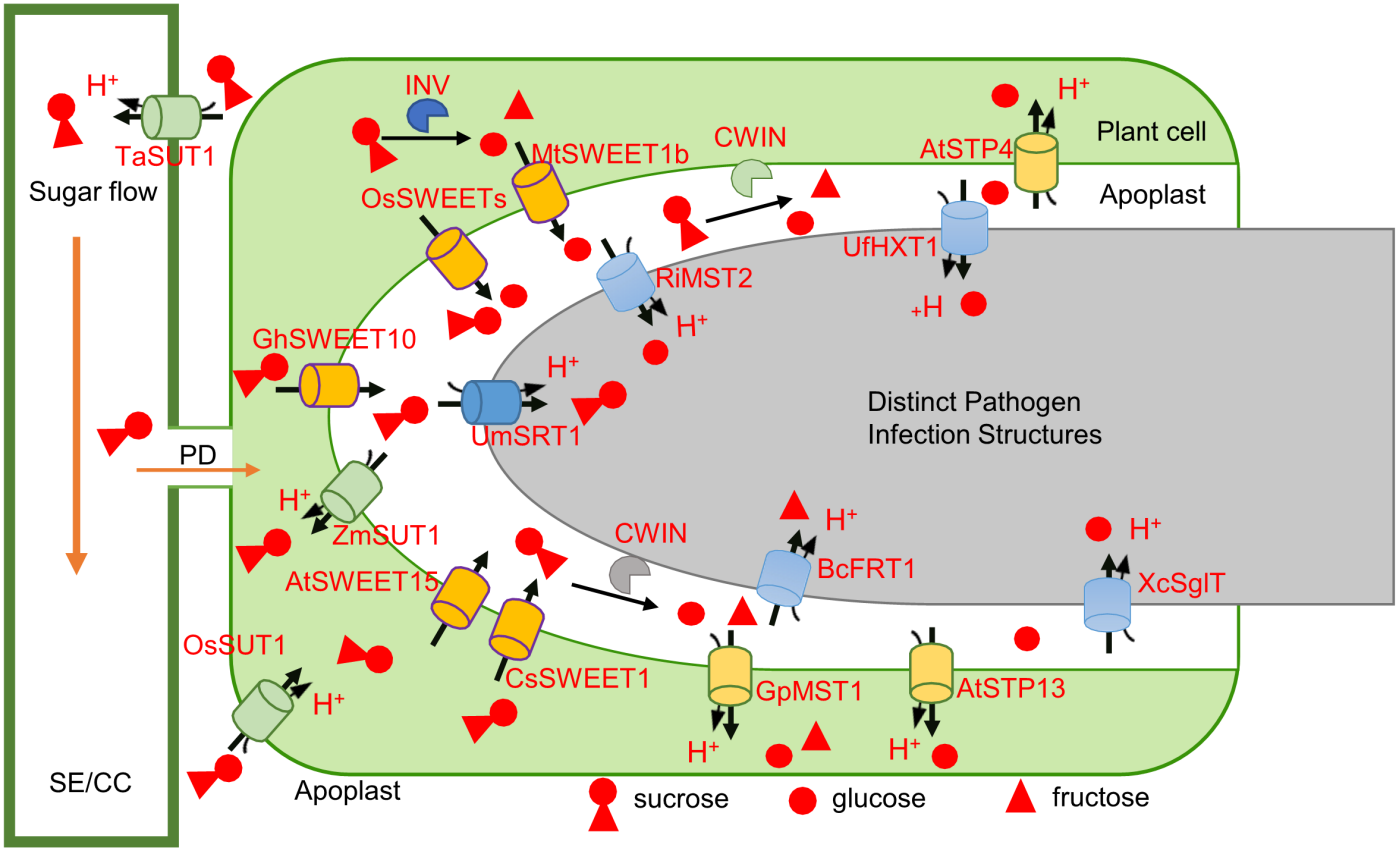
Sugar competition in plant-microbe interactions involves the participation of SWEETs, STPs, SUTs, and INVs of plants and pathogens. Once the pathogen successfully invades the host, the SWEET genes [OsSWEET2a (Gao et al., 2021), OsSWEET3a (Scofield et al., 2007), MtSWEET1b (An et al., 2019), OsSWEET11 (Gao et al., 2018), AtSWEET15 (Zhang et al., 2023), CsSWEET1 (Hu et al., 2014), and GhSWEET10 (Cox et al., 2017)] in the host are activated by the microbe target, and the INVs (CWINs) of the plant or microbe is induced, causing the sucrose (and glucose) in the host to flow out to the apoplast and partially hydrolyze into glucose and fructose (Proels and Hückelhoven, 2014; Liao et al., 2020). The extracellular sugar concentration therefore increases. Hexose is competitively absorbed by plant [AtSTP4 (Fotopoulos et al., 2003), AtSTP13 (Misra et al., 2019), and GpMST1 (Schüßler et al., 2006)] and pathogen [XcSglT (Zhang et al., 2023), BcFRT1 (Lemonnier et al., 2014), RiMST2 (Helber et al., 2011), and UfHXT1(Schuler et al., 2015)] MSTs, while sucrose is competing for the absorption by the plant [ZmSUT1 (Sosso et al., 2019), OsSUT1 (Scofield et al., 2007), and TaSUT1 (Aoki et al., 2014)] and microbe [UmSRT1 (Wahl et al., 2010)] sucrose transporters. Plants respond to the changes in the extracellular glucose concentration caused by microbial colonization to activate the immune response. The CWIN secreted by the microbe and the pathogenic sucrose absorption strategies removes the restriction imposed by the sugar leakage in plants.

Sugar is a key nutrient for which both plants and pathogens compete (Saddhe et al., 2021). Pathogens, irrespective of whether they are necrotrophic, biotrophic, or hemibiotrophic, compete for sugar with the host at the extracellular boundary formed between plants and pathogens (apoplast) during the establishment of the parasitic relationship (Naseem et al., 2017). The absorption of sugar by fungi and bacteria is mediated by sugar transporters, which differ according to the type of sugar absorbed, the way the sugar is ultimately utilized, and interactions with plant immunity, all of which are dependent on the varying sugar requirements of different pathogens. The competitive absorption of sugar generally includes two kinds of sugars: sucrose and hexose. The outcome of the competition is determined by characteristics and substrate specificities of sucrose and monosaccharide transporters in both host and pathogen. Therefore, investigation of the functions of proteins responsible for sugar transport in pathogens with different sugar preferences assists in the identification of immune-related regulatory pathways associated with pathogens with the same infection strategy and the overall spectrum of disease-resistance strategies. (**Figure 4**).

In the “sugar attack and defense” between plants and pathogens, plant sugar metabolism, signal transduction, and sugar transport proteins have received significant attention while the research on the sugar transport and metabolism of pathogens requires further investigation (Liu et al., 2022). More proteins related to sugar transport and metabolism of pathogenic bacteria require identification to improve the known regulatory networks of plant-pathogenic bacteria interaction, providing more direction for disease prevention and control. At present, the research on host sugar transporters reveals that pathogenic bacteria obtain sugar in plants *via* regulation of the host sugar transporters (such as SWEET) (Ji et al., 2022) and that some pathogenic bacteria avoid the host innate immune defense response caused by monosaccharide loss *via* sucrose absorption (Wahl et al., 2010). Therefore, inhibiting *SWEET* gene expression induced by pathogenic bacteria or increasing the expression level of plant transporters that mediate sugar recovery can be used as an effective strategy to improve plant disease resistance (Breia et al., 2021). AtSWEET2, located in vacuoles, reduces the availability of sugar released by vacuoles in the root after *Pythium* invasion, thereby increasing resistance (Chen et al., 2015). Therefore, the transport protein SWEET with its bidirectional transport function is crucial in the competition with pathogens for nutrients. In addition, *SWEET* genes play an important role in the process of phloem loading and unloading, specifically in the regulation of sugar distribution between source and sink, which is crucial for crop yield development (Braun, 2012). Consequently, the increase of plant resistance mediated by the SWEET mutation is accompanied by the reduction of crop yield. For example, the mutation of seed-filling related genes *OsSWEET4*, *11*, *15*, and *ZmSWEET4c* is accompanied by the reduction of grain weight of rice and maize (Gao et al., 2018; Li et al., 2021). There is a trade-off between yield and immunity mediated by SWEET proteins, which may be more fully understood as more SWEET gene functions are discovered.

The processes by which hosts and pathogens recognize each other are as important as the processes involved in competition. Previous studies have demonstrated changes in the expression of plant *CWIN* genes in response to signals of pathogen invasion, accompanied by local hydrolysis of sugars and promoting immune responses (Kocal et al., 2008). Glucose is not only a nutrient but also a signaling molecule that is recognized by sugar signaling receptors, such as HXT1 (Ehness et al., 1997; Patrick et al., 2013; Schuler et al., 2015). Therefore, the extracellular accumulation of hexose mediated by CWIN is the key factor in the induction of sugar-signaling immunity. The sugar-associated immune pathway involving the combination of monosaccharide transporter proteins for sugar recovery and receptor molecules for the recognition of hexose accumulation thus expands the immune regulatory network of the plant.

However, based on the recent research and according to the different modes of inducing plant susceptibility genes by different pathogens, the disease resistance of plants such as those with quantitative trait resistance (i.e., *Oryza sativa*) (Costanzo et al., 2011), can be enhanced to some extent by simultaneously combining multiple genes and providing more effective gene resources to direct broad-spectrum disease resistance breeding.

## Author contributions

YX, SY and GX designed this work, JC, YX, MS, RS and CZ wrote the manuscript. All authors contributed to the article and approved the submitted version.
